# Expression of the *Clonostachys rosea* lactonohydrolase gene by *Lactobacillus reuteri* to increase its zearalenone-removing ability

**DOI:** 10.1186/s12934-017-0687-8

**Published:** 2017-04-24

**Authors:** Wen-Chun Yang, Tsui-Chun Hsu, Kuan-Chen Cheng, Je-Ruei Liu

**Affiliations:** 1 Graduate Institute of Food Science and Technology, National Taiwan University, No. 1, Sec. 4, Roosevelt Rd., Taipei, 10617 Taiwan; 2 Department of Animal Science and Technology, National Taiwan University, No. 1, Sec. 4, Roosevelt Rd., Taipei, 10617 Taiwan; 3 Institute of Biotechnology, National Taiwan University, No. 1, Sec. 4, Roosevelt Rd., Taipei, 10617 Taiwan; 4 Department of Medical Research, China Medical University Hospital, China Medical University, No. 91, Hsueh-Shih Road, Taichung, 40402 Taiwan; 5 Agricultural Biotechnology Research Center, Academia Sinica, 128 Academia Road, Section 2, Nankang, Taipei, 11529 Taiwan

**Keywords:** Zearalenone, Lactonohydrolase, *Lactobacillus reuteri*, Probiotics

## Abstract

**Background:**

Mycotoxins are secondary metabolites produced by filamentous fungi that can contaminate agricultural crops in the field as well as during harvest, transportation, processing, or storage. Zearalenone (ZEN), a non-steroidal estrogenic mycotoxin, produced by *Fusarium* species, has been shown to be associated with reproductive disorders in farm animals and to a lesser extent in hyperoestrogenic syndromes in humans. Thus, the decontamination of ZEN in foods and feeds is an important issue.

**Results:**

In this study, the gene encoding ZHD101, a ZEN-degrading enzyme produced by *Clonostachys rosea* IFO 7063, was cloned into an *Escherichia coli*–*Lactobacillus* shuttle vector, pNZ3004, and the resultant plasmid pNZ-zhd101 was then introduced via electroporation into *Lactobacillus reuteri* Pg4, a probiotic strain isolated from the gastrointestinal tract of broilers. The transformed strain *L. reuteri* pNZ-zhd101 acquired the capacity to degrade ZEN. In addition, the production of recombinant ZHD101 did not affect cell growth, acid and bile salt tolerance, and had only a minor effect on the adhesion ability of *L. reuteri* pNZ-zhd101.

**Conclusions:**

To the best of our knowledge, this is the first report of successful expression of a ZEN-degrading enzyme by intestinal lactobacilli.

## Background

Mycotoxins are secondary metabolites produced by filamentous fungi that can contaminate agricultural crops in the field as well as during harvest, transportation, processing, or storage. The Food and Agriculture Organization (FAO) estimates that 25% of the world’s crop-based agricultural commodities are contaminated with mycotoxins, resulting in an estimated global loss of foodstuffs in the range of 1000 million tonnes annually [1]. Zearalenone (ZEN), a non-steroidal estrogenic mycotoxin produced by *Fusarium* fungi, is one of the most commonly found mycotoxins in food and feed [2]. ZEN can activate estrogenic receptors, resulting in reproductive disorders in farm animals and occasionally in hyperoestrogenic syndrome in humans [3]. Thus ZEN not only causes significant economic losses due to the lower efficacy of livestock production but also poses a health risk to humans who consume ZEN-contaminated foods [4]. The prevention of ZEN contamination in food and feed is an ideal solution for reducing the health rick of ZEN. However, it is considered that ZEN contamination cannot be avoided by the current agricultural practice [5]. Therefore, detoxification of ZEN in foods and feeds is another option for agricultural commodities already contaminated with ZEN. The strategies for decontamination of ZEN in foods and feeds include chemical methods such as exposure of ZEN-containing foods to ozone or hydrogen peroxide; physical methods such as extrusion processing; and biological methods such as using biotransforming agents to degrade ZEN into non-toxic metabolites or using adsorbing agents to decrease its bioavailability [3]. Among these ZEN detoxification methods, biological methods are preferable because they provides the opportunity for removal of mycotoxins under mild conditions without using harmful chemicals or causing significant losses in nutritive value and palatability of decontaminated food and feed [6, 7]. ZHD101, a lactonohydrolase produced by the fungal species *Clonostachys rosea*, converts ZEN into 1-(3,5-dihydroxy-phenyl)-10-hydroxy-1-undecen-6-one, which is a markedly less toxic product [8]. In previous studies, ZHD101 has been shown to be successfully expressed by *Escherichia coli*, *Saccharomyces cerevisiae*, and rice plants, and the recombinant ZHD101 produced by these genetically modified organisms effectively degraded ZEN [9, 10].

Probiotics have long been used as feed additives because of their abilities to normalize gut microbiota, boost the immune system, prevent diarrhea, and improve feed conversion efficiency [11, 12]. Although probiotics exert a number of beneficial effects, it has been speculated that their properties could be further improved through genetic modification [13, 14]. Examples of such genetically modified probiotics include those that produce antigens, enzymes, and cytokines for immune intervention [15, 16]. *Lactobacillus reuteri* is one of the potential probiotics that frequently occurs in the intestinal microflora of animals [17]. *L. reuteri* Pg4 was originally isolated from the gastrointestinal tract of a healthy broiler and has been shown to be capable of tolerating acid and bile salts, inhibiting pathogen growth, and adhering to mucin and mucus [18]. Moreover, *L. reuteri* Pg4 has been used to heterologously express some fibrolytic enzymes including β-glucanase, xylanase, and cellulase. Recombinant *L. reuteri* Pg4 strains have been demonstrated to acquire the capacity to break down fibers without losing their probiotic properties [14, 19, 20].

In the present study, we describe the heterologous expression of the *zhd101* gene derived from *C. rosea* in *L. reuteri* Pg4. We also examined the heterologous enzyme production, acid and bile salt tolerance, as well as the adherence capability of the transformed *L. reuteri* strain.

## Results

### Heterologous expression of ZHD101 in recombinant *L. reuteri* Pg4

**Fig. 1:**
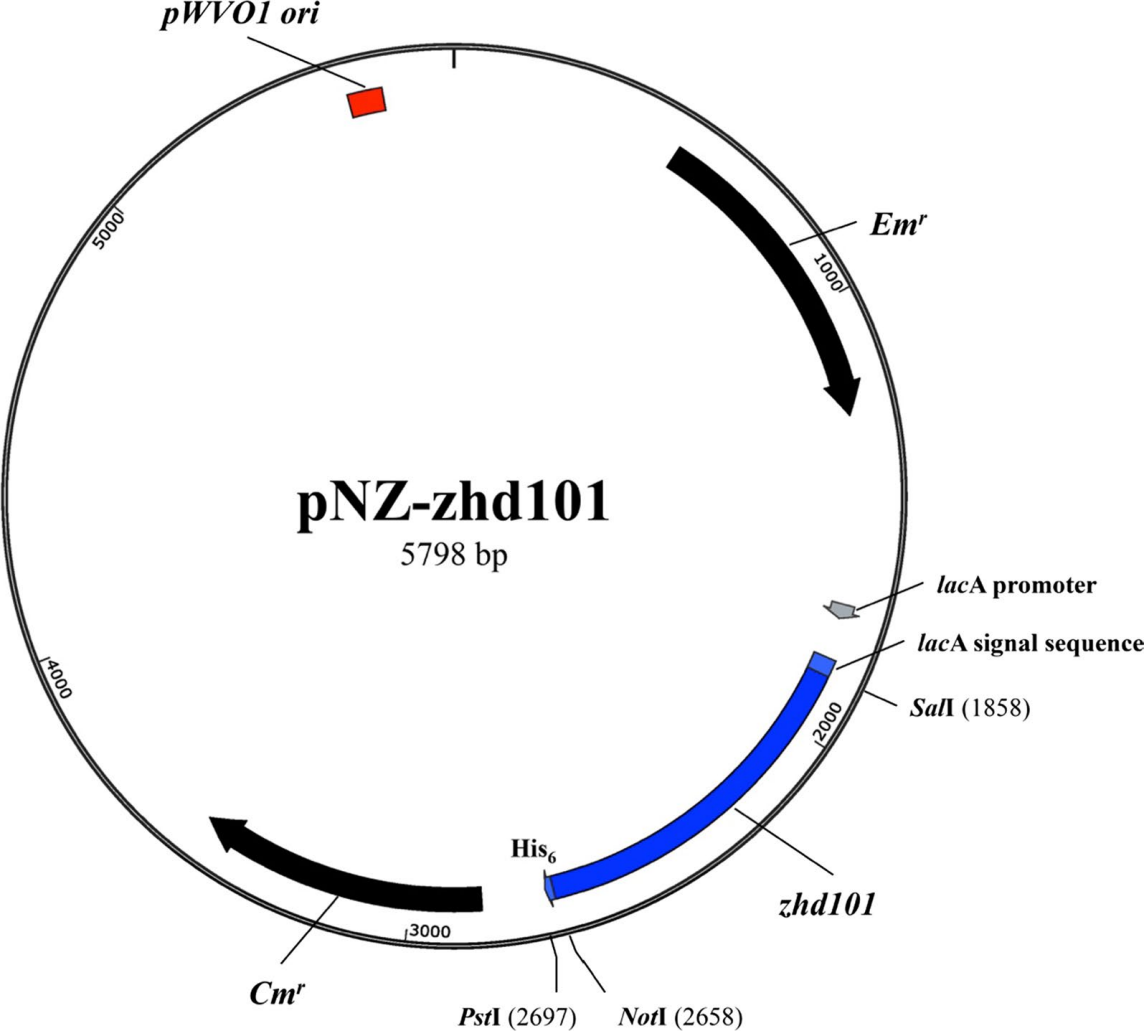
*Lactobacillus* expression plasmid harboring the *Clonostachys rosea* zearalenone hydrolase gene *zhd101*

**Fig. 2:**
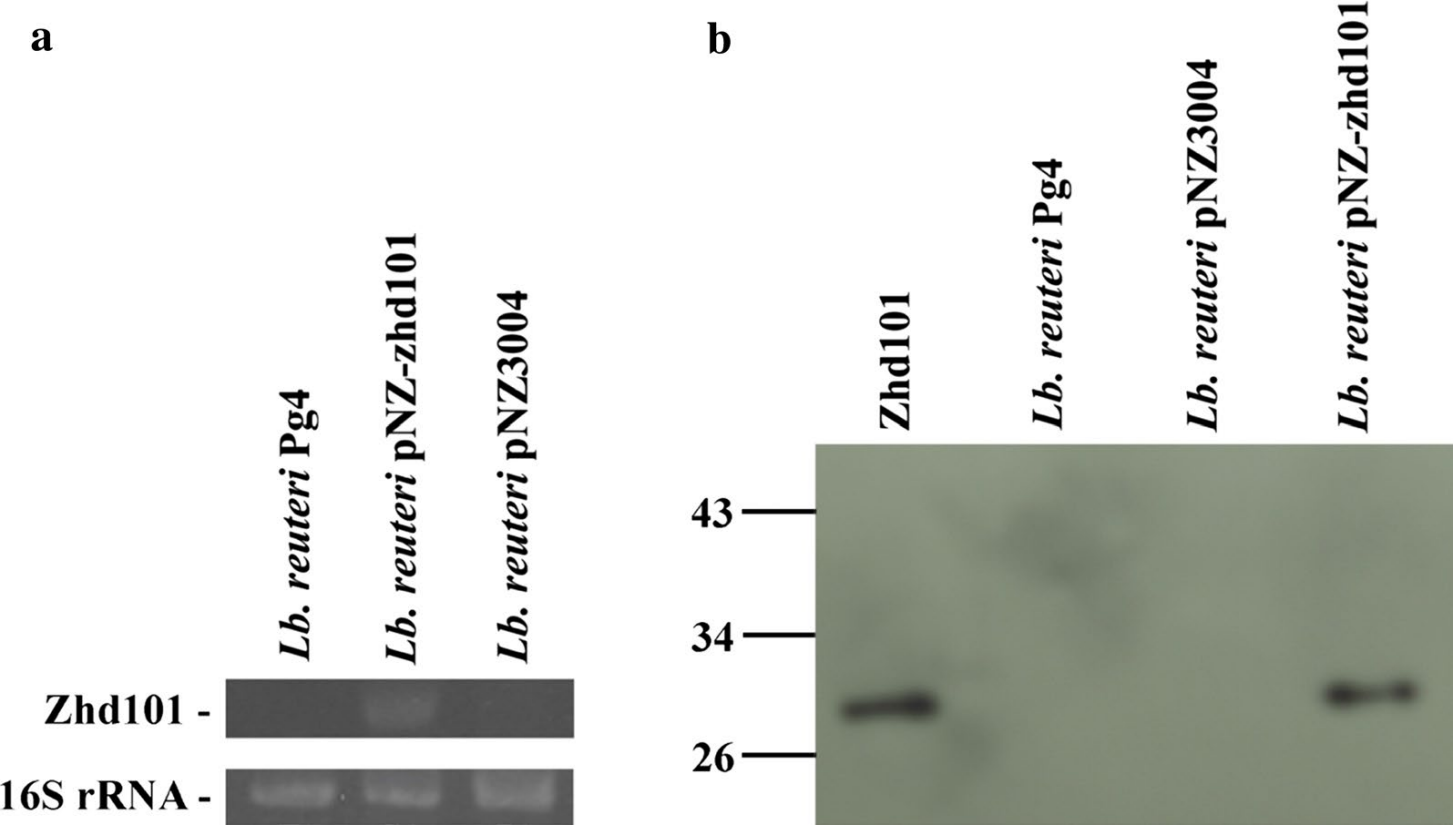
Expression of Zhd101 in *Lactobacillus reuteri* cells. **a** RT-PCR for Zhd101 in *Lb. reuteri* Pg4, *Lb. reuteri* pNZ-zhd101, and *Lb. reuteri* pNZ3004, along with the housekeeping gene 16S rRNA. **b** Western blot for purified recombinant Zhd101 and Zhd101 in the intracellular extracts of *Lb. reuteri* Pg4, *Lb. reuteri* pNZ3004, and *Lb. reuteri* pNZ-zhd101. The samples (2 μg of protein in each lane) were separated by SDS-PAGE with a 12.5% gel and probed with mouse polyclonal anti-zhd101 antibody and horseradish-peroxidase-linked anti-mouse IgG antibody as the primary and secondary antibodies, respectively

For heterologous expression of ZHD101 in *L. reuteri* Pg4, the DNA fragments encoding *C. rosea* ZHD101 were inserted into the *Lactobacillus* expression vector pNZ3004, resulting in the plasmid pNZ-zhd101 (Fig. 1). The plasmids pZN3004 and pNZ-zhd101 were then introduced via electroporation into *L. reuteri* Pg4. The transformation efficiency of pNZ-zhd101 was similar to that of pNZ3004 [(8–10) × 10^2^ transformants/μg of DNA]. The presence of the *zhd101* gene in *L. reuteri* pNZ-zhd101 was demonstrated by direct colony PCR (results not shown). The transcription of ZHD101 in *L. reuteri* pNZ-zhd101 was further confirmed by reverse transcription PCR (RT-PCR) analysis. A 0.8-kb fragment, which is consistent in size with *zhd101*, was amplified by RT-PCR from total RNA extracted from *L. reuteri* pNZ-zhd101 cells but was not detected from RNA extracted from *L. reuteri* Pg4 or *L. reuteri* pNZ3004 cells, indicating that ZHD101 RNA was successfully expressed by *L. reuteri* pNZ-zhd101 (Fig. 2a).

The translation of ZHD101 in *L. reuteri* Pg4 and its transformed strains was confirmed by western blot analysis using ZHD101-specific polyclonal antibodies. As shown in Fig. 2b, the antibodies clearly labeled a band at the expected size for ZHD101 (about 30 kDa) in the intracellular extract of *L. reuteri* pNZ-zhd101 cells, but not in extracts of *L. reuteri* Pg4 and *L. reuteri* pNZ3004 cells, indicating that ZHD101 could be heterologously expressed by *L. reuteri* pNZ-zhd101.

### Growth characteristics and ZEN-degrading activity of *L. reuteri* pNZ-zhd101

**Fig. 3:**
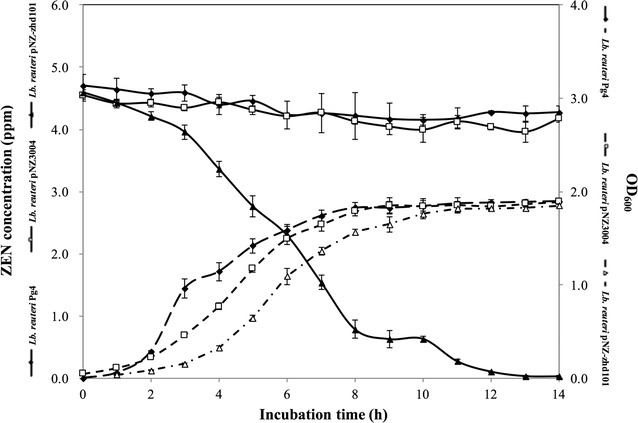
Changes in ZEN concentrations of MRS media containing ZEN (4.5 mg/L) incubated with *L. reuteri* Pg4 or *L. reuteri* pNZ-zhd101. The pH values of culture media were adjusted to pH 7.0 by adding 0.1 N NaOH every hour

To determine the ZEN-degrading activity, *L. reuteri* Pg4, *L. reuteri* pNZ3004, and *L. reuteri* pNZ-zhd101 were respectively inoculated into de Man, Rogosa and Sharpe (MRS) broth containing 4.5 mg/L of ZEN and allowed to ferment for 14 h. The pH of the broth was maintained at 7.0 during the fermentation. As shown in Fig. 3, *L. reuteri* Pg4 reached the stationary phase after 8 h of fermentation, with an OD_600_ of 1.83 ± 0.06 and bacterial counts of 6.65 ± 1.21 × 10^9^ CFU/mL. *L. reuteri* pNZ3004 also reached the stationary phase after 8 h of fermentation; however, it had a lower OD_600_ than *L. reuteri* Pg4 during the exponential phase. *L. reuteri* pNZ-zhd101 reached the stationary phase after 10 h of fermentation and had a lower OD_600_ than the other two strains during the exponential phase. Although *L. reuteri* pNZ-zhd101 took longer to reach the stationary phase, the cell counts of *L. reuteri* pNZ-zhd101did not differ significantly from those of *L. reuteri* Pg4 and *L. reuteri* pNZ3004 in the stationary phase.

The ZEN-degrading activity of *L. reuteri* pNZ-zhd101 was evaluated by determining the ZEN concentrations in MRS broth containing ZEN during the fermentation in a fermentor. As a reference and to compare ZEN-degrading activities, *L. reuteri* Pg4 and *L. reuteri* pNZ3004 were cultured and investigated using the same procedure. As shown in Fig. 3, *L. reuteri* pNZ-zhd101 displayed characteristic degradation effects on ZEN. After incubation of *L. reuteri* pNZ-zhd101 in the MRS broth containing 4.5 mg/L of ZEN for 14 h, the ZEN concentration decreased to 0.03 ± 0.01 mg/L, while that observed for *L. reuteri* Pg4 and *L. reuteri* pNZ3004 was 4.28 ± 0.10 and 4.21 ± 0.02 mg/L, respectively (Fig. 3). These results demonstrated that the ZEN-degrading activity of *L. reuteri* pNZ-zhd101 was significantly greater than the activities of *L. reuteri* Pg4 and *L. reuteri* pNZ3004, indicating that ZHD101 was functionally expressed by *L. reuteri* pNZ-zhd101.

### Acid and bile salt tolerance of *L. reuteri* pNZ-zhd101

**Fig. 4:**
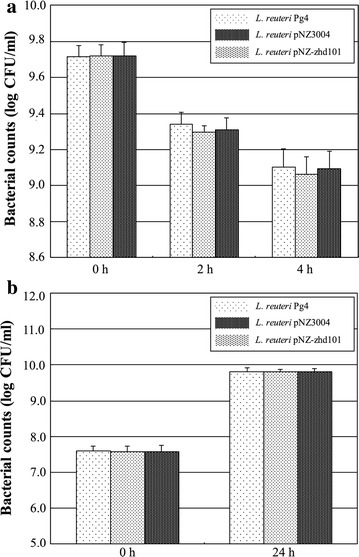
Survival of *L. reuteri* Pg4, *L. reuteri* pNZ3004 and *L. reuteri* pNZ-zhd101 after incubation at pH 3.0 (**a**) or in the presence of 0.5% ox gall (**b**). The *bars* represent standard errors of the means calculated from three independent experiments performed in triplicate

The acid tolerance of *L. reuteri* Pg4, *L. reuteri* pNZ3004, and *L. reuteri* pNZ-zhd101 was evaluated by culturing the bacterial cells at pH 3.0 and at 37 °C for 4 h, while the bile-salt tolerance of the *L. reuteri* strains was determined by culturing the bacterial cells in MRS broth containing 0.5% ox gall at 37 °C for 24 h. As shown in Fig. 4, all *L. reuteri* strains survived after an incubation period of 4 h at pH 3.0 (Fig. 4a) and after incubation for 24 h in MRS broth containing 0.5% ox gall (Fig. 4b). The bacterial counts of *L. reuteri* pNZ3004 and *L. reuteri* pNZ-zhd101 did not differ significantly from those of *L. reuteri* Pg4 in the acid condition or in the presence of bile salts. These results indicated that all of the *L. reuteri* Pg4 strains have the ability to tolerate acid and bile salts.

### Adhesion ability of *L. reuteri* pNZ-zhd101

**Fig. 5:**
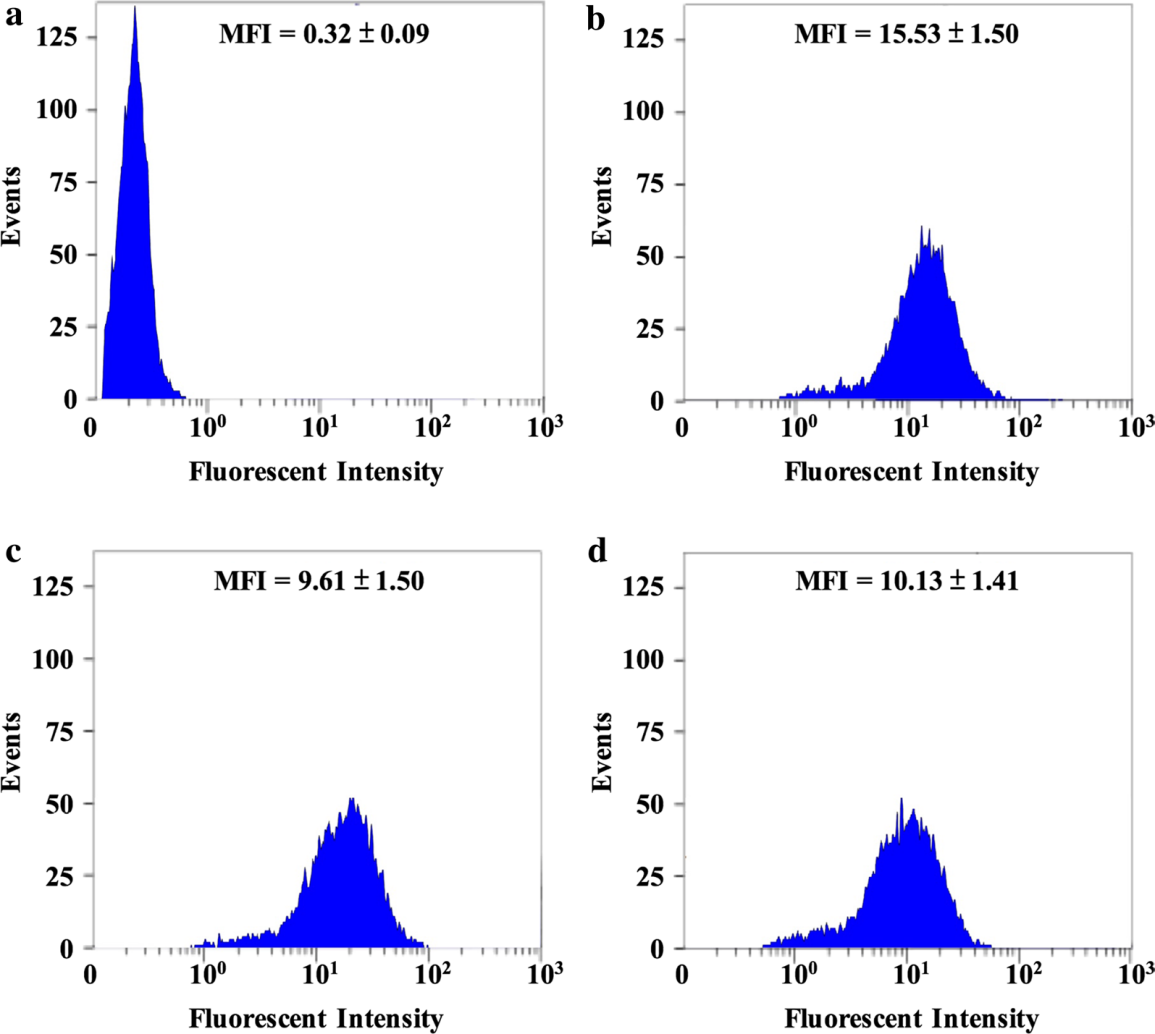
Flow cytometric analysis of *Lactobacillus* adherence to Caco-2 cells. **a** Autofluorescence of Caco-2 cells. **b** Caco-2 cells exposed to fluorescently labeled *L. reuteri* Pg4. **c** Caco-2 cells exposed to fluorescently labeled *L. reuteri* pNZ3004. **d** Caco-2 cells exposed to fluorescently labeled *L. reuteri* pNZ-zhd101. Bacterial cells were labeled with hexidium iodide (HI) and incubated with Caco-2 cells for 2 h. For each experiment, 10,000 Caco-2 cells were analyzed. The red fluorescence intensity of the Caco-2 cells with adherent lactobacilli was measured by flow cytometry at 620 nm emission wavelengths. Mean red fluorescence intensity (MFI) was calculated from two independent experiments performed in triplicate

The adhesion abilities of the *L. reuteri* strains were determined by culturing the hexidium iodide (HI)-stained *L. reuteri* cells with Caco-2 cells. The Caco-2 cells had weak autofluorescence as shown in the histogram plotted in Fig. 5a. After incubating Caco-2 cells with each HI-stained *L. reuteri* strains for 2 h, there was a shift along the fluorescence axis relative to the autofluorescence expressed by Caco-2 cells alone (Fig. 5b–d), indicating adhesion of bacterial cells to the Caco-2 cells. The mean fluorescence intensity of Caco-2 cells incubated with HI-stained *L. reuteri* Pg4 was significantly greater than that of Caco-2 cells alone (15.53 ± 1.50 *vs.* 0.42 ± 0.11) (*p* < 0.05), indicating that *L. reuteri* Pg4 efficiently adhered to Caco-2 cells (Fig. 5b). The mean fluorescence intensity of Caco-2 cells incubated with *L. reuteri* pNZ-zhd101 (10.13 ± 1.41) was also significantly greater than that of Caco-2 cells alone (*p* < 0.05) but was significantly lower than that of Caco-2 cells incubated with *L. reuteri* Pg4, indicating that constitutive expression of the recombinant ZHD101 protein in *L. reuteri* pNZ-zhd101 resulted in a decrease in adhesion ability to Caco-2 cells. However, *L. reuteri* pNZ-zhd101 retained its adhesion ability at a certain level.

## Discussion

Probiotics exert beneficial effects on animal performance by normalizing gastrointestinal microflora and offer great potential as feed additives to replace antibiotics [21]. Genetic modification could increase the effectiveness and usefulness of some probiotics. For example, certain probiotics could be modified to express and secrete of specific heterologous enzymes [16]. Probiotics that secrete specific enzymes can provide additional beneficial effects by delivering the enzymes directly to the target site, typically the intestine, where most of the feed digestion and nutrient absorption occurs. Other benefits include the reduced cost of enzyme supplementation because the genes for these enzyme genes could be replicated and expressed by the probiotics within the digestive tract [20]. In previous studies, we induced *L. reuteri* Pg4 to express fibrolytic enzymes and found that the transformed *L. reuteri* strains acquired the capacity to break down plant fibers, resulting in body-weight gain and improved feed-conversion efficiency in broilers [14, 19, 22, 23]. In the present study, the *C. rosea zhd101* gene was introduced into *L. reuteri* Pg4 and the transformed strain *L. reuteri* pNZ-zhd101 successfully expressed ZHD101 and acquired the capacity to degrade ZEN. To the best of our knowledge, this is the first report of successful expression of a ZEN-degrading enzyme by intestinal lactobacilli.

After incubating the three *L. reuteri* strains in MRS broth containing 4.5 mg/L of ZEN for 14 h, we found that the concentration of ZEN in *L. reuteri* pNZ-zhd101-containing broth decreased by 99.3%. In contrast, the ZEN concentration in broth containing *L. reuteri* Pg4 had only decreased by 9.1% and that in broth containing *L. reuteri* pNZ3004 only decreased by 7.5% (Fig. 2). Although the reduction in ZEN concentration by *L. reuteri* Pg4 and *L. reuteri* pNZ3004 was significantly lower than the reduction by *L. reuteri* pNZ-zhd101, the result shows that *L. reuteri* Pg4 and *L. reuteri* pNZ3004 have ZEN-removing abilities. Previous studies have shown that some probiotics such as *Bacillus, Bifidobacterium*, *Lactococcus*, and *Lactobacillus* strains have mycotoxin-adsorption abilities [24, 25]. The ZEN-adsorption ability of *L. rhamnosus* was attributed to its polysaccharide components of the cell wall [26] while the fumonisin B1-adsorption ability of *B. subtilis* was attributed to its peptidoglycans [27]. In this study, *L. reuteri* Pg4 and *L. reuteri* pNZ3004 only slightly decreased the concentration of ZEN in culture media, suggesting that *L. reuteri* Pg4 may possess ZEN-adsorption ability. Future research will be conducted to demonstrate the ZEN-adsorption ability of *L. reuteri* Pg4 and confirm which components contribute to the adsorption of ZEN.

Takahashi-Ando et al. found that recombinant ZHD101 obtained from an *E. coli* expression system was irreversibly inactivated at pH values below 4.5 [9]. For this reason, we maintained the pH value of the culture medium for *L. reuteri* pNZ-zhd101 at 7.0 during the fermentation process and found that *L. reuteri* pNZ-zhd101 could effectively remove ZEN under this culture condition (Fig. 2). These results indicated that the recombinant ZHD101 produced by *L. reuteri* pNZ-zhd101 is in an active form at pH 7.0. The mean pH values in small intestine, caecum, and colon of swine are 6.5, 6.1, and 6.5, respectively [28], while the mean pH values in crop, small intestine, caecum, and colon of chickens are 6.1, 6.4, 6.4, and 6.6, respectively [29]. Since the intestinal environments are mildly acidic to neutral, we believe that the recombinant ZHD101 produced by *L. reuteri* pNZ-zhd101 can be directly delivered to the intestinal tract in an active form.

Probiotics must be able to resist to the digestion process in the gastrointestinal tract [11]. The most difficult hurdles associated with the survival of the probiotics in the gastrointestinal tract are the acidic conditions of the stomach and the bile salts in the duodenum. Gastric acid in stomach represents a primary defense mechanism against the majority of ingested microorganisms, while the bile salts in the duodenal section of the small intestine reduce the survival of bacteria because the lipids and fatty acids comprising the bacterial cell membranes are very susceptible to destruction by bile salts [30]. In a previous study, we observed that *L. reuteri* Pg4 exhibits resistance to acidic conditions and contact with bile salts in vitro [18]. In this study, all three *L. reuteri* Pg4 strains survived after an incubation period of 4 h at pH 3.0 (Fig. 3a) or 10 h in MRS broth containing 0.5% ox gall (Fig. 3b). Moreover, the bacterial counts of the *L. reuteri* transformed strains did not differ from those of *L. reuteri* Pg4 in the acid condition or in the presence of bile salts. These results indicated that *L. reuteri* Pg4 strains have the potential to survive transit through the stomach and might be able to survive in the intestinal environment in where they can effectively work.

After passing through the stomach and surviving in the intestinal tract, probiotics must be able to adhere to the intestinal mucosal in order to extend their maximum probiotic effects [11]. *L. reuteri* is a gut symbiont that normally colonizes in the upper intestinal tract in animals and is a stable part of colonic microbiota in humans [31]. Thus *L. reuteri* strains usually show a great ability to adhere to mucosal cells [32–34]. A previous study demonstrated that 40% of the *Lactobacillus* cells randomly isolated from the digest of the ileum and caecum of broiler chickens fed with a recombinant *L. reuteri* strain possessed the capability of secreting recombinant proteins, indicating that *L. reuteri* administered orally could colonize the intestinal tract [22]. The Caco-2 cell line has been used extensively for in vitro evaluation of adhesion capacity of probiotics [20, 34, 35]. In this study, Caco-2 cells that had been incubated with fluorescent-labeled *L. reuteri* pNZ-zhd101 for 2 h showed a shift along the fluorescence axis when compared with the autofluorescence expressed by Caco-2 cells alone (Fig. 4), indicating that the *L. reuteri* cells adhered to the Caco-2 cells. Probiotics can compete with pathogenic bacteria for the same binding sites on intestinal epithelial cells [36]. In addition, the specific enzymes secreted by recombinant probiotics that have attached themselves to intestinal epithelial cells could react directly on the substrate present in the digesta in the small intestine, where the majority of ZEN is absorbed [37]. Therefore, the ability of *L. reuteri* pNZ-zhd101 to adhere to intestinal mucosal cells extends its residence time in the intestinal tract and, hence allows it to achieve its maximum probiotic effects. Several exported proteins produced by *L. reuteri* have been reported to mediate the adhesion of bacterial cells to the intestinal epithelial cells [34]. The collagen-binding protein Cnb is one of the reported extracellular adhesion proteins produced by *L. reuteri*. Previous studies demonstrated that Cnb mediated *L. reuteri* adhesion to the intestinal epithelial cells and could be used as the anchor proteins for display of heterologous proteins on the cell surface of *L. reuteri* [20, 34]. The transformed *L. reuteri* strain not only produced heterologous proteins on its cell surface but also showed higher adhesion ability than the parental *L. reuteri* strain. In order to increase the adhesion ability of *L. reuteri* pNZ-zhd101 and extend the residence time of ZHD101 in the intestinal tract, future research will focus on Cnb as the anchor protein for display of ZHD101 on the cell surface of *L. reuteri* Pg4.

## Conclusion

We successfully cloned the *C. rosea* lactonohydrolase gene *zhd101* in a *L. reuteri* strain and demonstrated that the heterologous ZHD101 was functionally expressed by the transformed strain *L. reuteri* pNZ-zhd101. The production of heterologous ZHD101 did not affect cell growth, acid and bile salt tolerance, and had only a minor effect on the adhesion ability of *L. reuteri* pNZ-zhd101. Therefore, we suggested that *L. reuteri* pNZ-zhd101 has a potential to be used as a probiotic feed additive for degradation of ZEN.

## Materials

### Chemicals and reagents

A stock solution of ZEN was prepared by dissolving the solid powder (Sigma-Aldrich Co. St. Louis, MO) in acetonitrile (0.5 mg/mL) and was stored in the dark at −20 °C. For high-performance liquid chromatography (HPLC) calibration or spiking purposes, the ZEN standard solutions were brought to room temperature before use and were prepared freshly by diluting the stock solutions in methanol. Acetonitrile and methanol (HPLC grade) were obtained from J. T. Baker Inc. (Phillipsburg, NJ). Water for the HPLC mobile phase was purified successively by reverse osmosis and a Milli-Q system (Millipore, Bedford, MA). All other chemicals used were analytical reagent grade and purchased from Sigma-Aldrich (St. Louis, MO). All solutions prepared for HPLC were filtered through a 0.22 μm nylon filter before use.

### Plasmids, bacterial strains, cell line, and culture conditions

The *Lactobacillus* expression vector pNZ3004 [38] was transformed into *L. reuteri* Pg4 to express recombinant ZHD101 proteins [18]. The *Escherichia coli* expression vector pET-46 Ek/LIC (Novagen, Madison, WI) was transformed into *E. coli* BL21 (DE3) (Novagen) to overexpress the recombinant ZHD101 protein. The pGEM-T Easy vector (Promega, Madison, WI) was used for all subcloning procedures and *E. coli* DH5α (Invitrogen, Carlsbad, CA) was used as the host. *Lactobacillus* strains were grown in MRS broth (Difco Laboratories, Detroit, MI) at 37 °C without shaking. *E. coli* was cultured in Luria-Bertani (LB) broth (Difco Laboratories) at 37 °C in an orbital shaker at 250 rpm. Agar plates were prepared by adding agar (1.5% w/v) (Difco Laboratories) to the broth. The cancer-derived human colonic intestinal epithelial cell line Caco-2 was used for the in vitro assay of bacterial adhesion to mammalian epithelial cells. Caco-2 cells were purchased from the Bioresource Collection and Research Center (Hsinchu, Taiwan) and were routinely grown in Dulbecco’s Modified Eagle Medium (Biological Industries, Bet-Haemek, Israel) supplemented with 4 mM l-glutamine (Biological Industries), 1.5 g/L sodium bicarbonate (Biological Industries), 4.5 g/L glucose (Sigma-Aldrich Co., St Louis, MO), 10 mg/L human transferrin (Sigma-Aldrich Co.), and 10% fetal calf serum (Moregate Biotech, Queensland, Australia) at 37 °C under a humidified atmosphere of 95% air and 5% CO_2_.

### DNA isolation and manipulation

Plasmid DNA was isolated from *E. coli* using the QIAprep Miniprep Kit (Qiagen Inc.) and plasmid DNA was isolated from *L. reuteri* according to the method described by O’Sullivan and Klaenhammer [39]. Restriction enzymes and T4 DNA ligase (New England BioLabs Inc., Beverly, MA) were used in the subcloning procedures according to the manufacturer’s instructions. All other DNA manipulations were performed according to the established procedures [40]. All DNA sequences were determined by an automated sequencing service provided by Genomics Biotech Inc. (Taipei, Taiwan). Competent *E. coli* cells and *L. reuteri* Pg4 cells were prepared according to the methods described by Green et al. [40] and Hsueh et al. [35], respectively.

### Preparation of polyclonal antibody specific for ZHD101

The DNA fragments encoding *C. rosea* ZHD101 (GenBank accession number AB076037) were synthesized chemically and cloned into the vector pUC57 (Mission Biotech Inc., Taipei, Taiwan), resulting in the plasmid pUC57-zhd101. The DNA fragments of *zhd101* were amplified by PCR from pUC57-zhd101 using the oligonucleotide forward primer zhd-F1: 5′ GACGACGACAAGATGCGTACTCGTAGCACTAT 3′ and reverse primer zhd-R1: 5′ GAGGAGAAGCCCGGTTAAAGGTGTTTCTGAGTAG 3′ (the underlined sequences in the primers are additional sequences that are compatible with the ligation-dependent cloning (LIC) site of the *E. coli* expression vector pET-46 Ek/LIC) and annealed to pET-46 Ek/LIC according to the manufacturer’s instructions. The resultant plasmid, designated pET-zhd101, was used to transform *E. coli* BL21 (DE3). Overexpression and purification of the recombinant ZHD101 were performed by standard techniques [40]. The purified ZHD101 was used to prepare polyclonal antibodies in mice as a service provided by LTK Biolab Inc. (Hsinchu, Taiwan).

### Heterologous expression of zhd101 gene by *L. reuteri*

To subclone the *zhd101* gene into the *Lactobacillus* expression vector, the *zhd101* gene was amplified by PCR from pET-zhd101 using the oligonucleotide forward primer zhd-F2: 5′ GTCGACAATGCGTACTCGTAG 3′ and reverse primer zhd-R2: 5′ CTGCAGTCAAAGGTGTTTCT 3′ (the underlined sequences in the primers are additional sequences that represent the restriction sites for *Sal*I and *Pst*I, respectively). The PCR fragments encoding ZHD101 were digested with *Sal*I and *Pst*I, and ligated with *Sal*I-*Pst*I-digested pNZ3004 to generate pNZ-zhd101. The pNZ3004 and pNZ-zhd101 plasmids were individually transformed via electroporation into *L. reuteri* Pg4 according to the method described by Hsueh et al. [35]. The transformants, designated *L. reuteri* pNZ3004 and *L. reuteri* pNZ-zhd101, were then confirmed not only by direct colony PCR using the zhd-F2 and zhd-R2 primer set but also by RT-PCR and western blot analyses.

For the RT-PCR analysis, *Lb. reuteri* Pg4, *L. reuteri* pNZ3004, and *L. reuteri* pNZ-zhd101 were transferred to MRS broth and incubated statically at 37 °C for 24 h. The cells were harvested by centrifugation at 5000×*g* for 20 min at 4 °C. Total RNA was extracted from the lactobacilli cells and the transcription of the *zhd101* gene was analyzed by RT-PCR using the zhd-F2 and zhd-R2 primer set according to the methods described by Hsueh et al. [34]. The transcription of the housekeeping 16S rRNA gene was analyzed in parallel for each sample using the 16S-27f (5′ AGAGTTTGATCMTGGCTCAG 3′) and 16S-1492r (5′ CGGTTACCTTGTTACGACTT 3′) primer sets.

For the Western blot analysis, *Lb. reuteri* Pg4, *L. reuteri* pNZ3004, and *L. reuteri* pNZ-zhd101 cells were cultivated and harvested as described above. The cell pellet was resuspended in 0.1 M phosphate-buffered saline (PBS; pH 7.4) and then sonicated for 10 min with an ultrasonicator (Model XL, Misonix, Farmingdale, NY). After centrifugation at 13,000×*g* for 20 min at 4 °C, the intracellular extract was collected and analyzed using sodium dodecyl sulfate polyacrylamide gel electrophoresis (SDS-PAGE) according to the method described by Laemmli [41]. After electrophoresis, the proteins were transferred to a polyvinylidene difluoride (PVDF) membrane to perform the Western blot analysis according to the methods described by Huang et al. [20]. The primary and secondary antibodies used in the analysis were mouse polyclonal anti-zhd101 antibody (1:10 dilution in PBS, LTK Biolab. Inc.) and horseradish-peroxidase-linked anti-mouse IgG antibody (1:10 dilution in PBS, GE Healthcare, Piscataway, NJ), respective.

### Degradation of ZEN by *L. reuteri* strains

Degradation of ZEN by the *L. reuteri* strains was performed in a 5-L fermentor with a 2.0-L working volume. The fermentor with MRS medium was autoclaved at 121 °C and 1.5 atm pressure for 15 min. The stock solutions of ZEN and erythromycin were sterilized by filtering them through a 0.22-μm nylon filter. After autoclaving, ZEN was added to the fermentor to reach a final ZEN concentration of 4.5 mg/L. For the culturing of *L. reuteri* pNZ3004 and *L. reuteri* pNZ-zhd101, erythromycin was added to the fermentor to reach a final erythromycin concentration of 10 mg/L. The fermentor was then inoculated with 1% (v/v) of an overnight culture of *L. reuteri* Pg4, *L. reuteri* pNZ3004, or *L. reuteri* pNZ-zhd101 and incubated at 37 °C for 14 h by gently stirring at 250 rpm. To maintain the pH of the culture medium at 7.0, sodium hydroxide (4.0 N) and hydrochloric acid (3.0 N) were added to the fermentor automatically. During the incubation period, samples were taken every hour to quantify ZEN concentration using HPLC, to determine viable cell count using the standard agar plate method, and to estimate the stage of the cultured cell population by measuring turbidity at 600 nm (OD_600_).

### Determination of ZEN concentration by HPLC

The ZEN concentration in the samples was determined by HPLC according to the method described by Yi et al. [7]. The HPLC analysis was performed on a LC-20 AT delivery system (Shimadzu, Kyoto, Japan) equipped with a RF-10AXL fluorescence detector (Shimadzu), a Shimpack CLC-ODS column (Shimadzu; 250 × 4.6 mm i.d., particle size 5 μg), and a SIL-10ADVP AutoSampler (Shimadzu). Before HPLC analysis for ZEN, 1 mL of sample was mixed with 4 mL of acetonitrile–water (84:16, v/v with ultrapure and deionized water) and then agitated on an orbital shaker at 180 rpm for 90 min. After centrifugation at 13,000 rpm for 5 min, the acetonitrile phase was collected and cleaned up by using the Romer Mycosep 224 column (Romer Labs Inc., Union, MO) according to the manufacturer’s instructions. After evaporating to dryness under a stream of nitrogen at 60 °C, the dried residue was re-dissolved in 300 μL of methanol solution (80:20, v/v with ultrapure and deionized water), filtered through a 0.45-μm nylon filter to remove insoluble material, and then 20 μL of the resulted solution was injected into the HPLC to quantify the ZEN concentration. The mobile phase was a methanol solution (80:20, v/v with ultrapure and deionized water) and was eluted at a flow rate of 0.5 mL/min. and detection was performed at excitation and emission wavelengths of 225 and 465 nm, respectively.

### Acidic tolerance of the *L. reuteri* strains

Acid tolerance of the *L. reuteri* strains was determined according to the method by Liu et al. [14]. A 1-mL aliquot of the overnight culture of the *L. reuteri* strains was centrifuged at 5000×*g* for 10 min at 4 °C, the pellets were washed twice in sterile PBS (100 mM, pH 7.4) and then resuspended in 1 mL of the same buffer. The bacterial cells were then diluted 1/100 in pH-adjusted MRS broth (pH 3.0) and incubated statically at 37 °C for 4 h. During the incubation period, a 0.1-mL aliquot of sample was taken at 0, 2, and 4 h respectively to count the cell numbers using the standard agar plate method.

### Bile salt tolerance of the *L. reuteri* strains

Bile salt tolerance of the *L. reuteri* strains was determined as previously described by Liu et al. [14]. Briefly, a 1-mL aliquot of overnight culture of the *L. reuteri* strains was inoculated into 100 mL of MRS broth containing 0.5% ox gall (Sigma), and incubated statically at 37 °C for 24 h. During the incubation period, a 0.1-mL aliquot of sample was taken at 0 and 24 h respectively to count the cell numbers using the standard agar plate method.

### Adhesion abilities of the *L. reuteri* strains

Before the adhesion assays, Caco-2 cells were incubated with antibiotic-free medium at 37 °C for 24 h. After incubation, the Caco-2 cells were removed from tissue culture flasks with an EDTA-trypsin solution, and then washed three times with 100 mM PBS (pH 7.4). The Caco-2 cells were then resuspended in PBS containing mouse anti-human CD29 (integrin beta 1) monoclonal antibody conjugated with PerCP-eFluor 710 (diluted 1:10 in PBS; eBioscience. Inc., San Diego, CA) and incubated at room temperature for 1.5 h. After washing three times with PBS, the immunostained Caco-2 cells were resuspended in PBS and adjusted to a concentration of 1 × 10^6^ cells/mL.

*Lactobacillus reuteri* Pg4, *L. reuteri* pNZ3004, and *L. reuteri* pNZ-zhd101 cells were cultivated, harvested, and resuspended in 1 mL of 100 mM PBS (pH 7.4) as described above. They were then labeled with HI using the Live BacLight Bacterial Gram Stain Kit (Molecular Probes, Eugene, OR) according to the protocols provided by the manufacturer. Fluorescently labeled lactobacilli were added to the Caco-2 suspension to yield a final concentration of 10^8^ CFU/mL of lactobacilli and 10^6^ cells/mL of Caco-2 cells. After incubating at 37 °C for 2 h, the Caco-2 cells were harvested by centrifugation at 5000×*g* for 20 min at 4 °C and were washed three times with PBS to remove non-adherent lactobacilli. The fluorescence intensity of the Caco-2 cells with adherent lactobacilli was measured using the Cytomics FC500 Flow Cytometry System (Beckman Coulter, Inc.) with a 488-nm argon laser and a 620-nm emission filter for HI detection and a 755-nm emission filter for PerCP-eFluor 710 detection. Flow cytometry was gated to include Caco-2 cells and to exclude cellular debris and non-adherent bacteria using a dot plot displaying forward scatter (FSC) vs. side scatter (SSC). Cells inside the gated area were confirmed by staining with CD29 antibody conjugated with PerCP-eFluor 710. For each analysis, 10,000 events were acquired and the flow cytometric data obtained were analyzed using CXP software (Beckman Coulter, Inc.) to produce the histograms of each individual particle sample and to calculate the mean red fluorescence intensity for each cell population. Counts were made in triplicate for each procedure.

### Statistical analysis

All results were analyzed using the general linear-model procedure available with Statistical Analysis System software version 8.1 (SAS Institute Inc., Cary, NC). The Duncan’s multiple range test [42] was used to detect differences between treatment means. Each experiment was conducted in triplicate and repeated three times.

## Abbreviations

HI: hexidium iodide
HPLC: high-performance liquid chromatography
MRS: de Man, Rogosa and Sharpe
LB: Luria–Bertani
LIC: ligation-dependent cloning
PBS: phosphate-buffered saline
PVDF: polyvinylidene difluoride
SDS-PAGE: sodium dodecyl sulfate polyacrylamide gel electrophoresis
RT-PCR: reverse transcription PCR
ZEN: zearalenone
