# Erratum to: Expression of the *Clonostachys rosea* lactonohydrolase gene by *Lactobacillus reuteri* to increase its zearalenone-removing ability

**DOI:** 10.1186/s12934-017-0714-9

**Published:** 2017-06-12

**Authors:** Wen-Chun Yang, Tsui-Chun Hsu, Kuan-Chen Cheng, Je-Ruei Liu

**Affiliations:** 1 Graduate Institute of Food Science and Technology, National Taiwan University, No. 1, Sec. 4, Roosevelt Rd., Taipei, 10617 Taiwan; 2 Department of Animal Science and Technology, National Taiwan University, No. 1, Sec. 4, Roosevelt Rd., Taipei, 10617 Taiwan; 3 Institute of Biotechnology, National Taiwan University, No. 1, Sec. 4, Roosevelt Rd., Taipei, 10617 Taiwan; 4 Department of Medical Research, China Medical University Hospital, China Medical University, No. 91, Hsueh-Shih Road, Taichung, 40402 Taiwan; 5 Agricultural Biotechnology Research Center, Academia Sinica, 128 Academia Road, Section 2, Nankang, Taipei, 11529 Taiwan

## Erratum to: Microb Cell Fact (2017) 16:69 DOI 10.1186/s12934-017-0687-8

The original version of this article [1] contained the following errors that, unfortunately, could not be corrected in time before online publication.

1. The ‘Adhesion ability of *L. reuteri* pNZ-zhd101’ of the ‘Results’ section erroneously read: “The mean fluorescence intensity of Caco-2 cells incubated with HI-stained *L. reuteri* Pg4 was significantly greater than that of Caco-2 cells alone (15.53 ± 1.50 vs. 0.42 ± 0.11)…”.
  The sentence should instead read: “The mean fluorescence intensity of Caco-2 cells incubated with HI-stained *L. reuteri* Pg4 was significantly greater than that of Caco-2 cells alone (15.53 ± 1.50 vs. 0.32 ± 0.09)”.
2. The fourth paragraph of the ‘Discussion’ section erroneously read: “In this study, all three *L. reuteri* Pg4 strains survived after an incubation period of 4 h at pH 3.0 or 10 h in MRS broth containing 0.5% ox gall.
  The sentence should instead read: “In this study, all three *L. reuteri* Pg4 strains survived after an incubation period of 4 h at pH 3.0 or 24 h in MRS broth containing 0.5% ox gall”.
3. The second and third paragraphs of the ‘Discussion’ section erroneously cited Fig. 2. The correct citation is to Fig. 3. As a result, all subsequent figure citations were also incorrect and had to be renumbered.

The above errors have been corrected in the original article.
